# Treosulfan–fludarabine conditioning in infants with severe combined immunodeficiencies: Extended study of the UK paediatric treosulfan study

**DOI:** 10.1111/bjh.70453

**Published:** 2026-04-10

**Authors:** Su Han Lum, Sinéad Greener, Irum Latif Memon, Persis Amrolia, Zohreh Nademi, Robert Chiesa, Juliana Silva, Helen Young, Stephen Owens, Eleri Williams, Khuen Foong Ng, Terry Flood, Andrew R Gennery, Sophie Hambleton, Kanchan Rao, Mary Slatter

**Affiliations:** ^1^ Translational & Clinical Research Institute Newcastle University Newcastle upon Tyne UK; ^2^ Children's Haematopoietic Stem Cell Transplant Unit Great North Children's Hospital Newcastle Upon Tyne UK; ^3^ Bone Marrow Transplant Unit Great Ormond Street Hospital for Children NHS Foundation Trust London UK

**Keywords:** fludarabine, HSCT, SCID, treosulfan

## Abstract

Allogeneic haematopoietic stem cell transplantation (HSCT) is a curative therapy for severe combined immunodeficiency (SCID). Conditioning improves donor engraftment and freedom from immunoglobulin replacement (IgR) but increases the risks of acute and late toxicity. Treosulfan, a reduced toxicity alkylating agent, has emerged as an alternative to busulfan. In this UK multicentre study, we evaluated outcomes of 104 infants with SCID who underwent first HSCT following treosulfan‐fludarabine conditioning between 2006 and 2022. After a median follow‐up of 5.4 years, 5‐year overall survival (OS) and event‐free survivals (EFS) were 81% and 77% respectively. On multivariate analysis, molecularly undefined SCID (OS hazard ratio [HR] 5.61; EFS HR 5.55) and pre‐HSCT cytomegalovirus (CMV) infection (OS HR 3.94; EFS 3.68) were independently associated with inferior OS and EFS; RAG‐DCLRE1C genotypes also predicted worse EFS (HR 4.35). Cumulative incidence of endothelial cell dysfunction (ECD) was 11%. Treosulfan dose was not associated with OS, EFS, ECD or donor myeloid chimerism. Low mixed donor myeloid chimerism was observed across all treosulfan doses, but IgR freedom was achieved in 92% of survivors after first HSCT. Treosulfan‐fludarabine provides excellent survival with low endothelial toxicity for SCID HSCT, with potential for optimisation via pharmacokinetic guided dosing.

## INTRODUCTION

Severe combined immunodeficiencies (SCIDs) are rare monogenic disorders causing profound defects in lymphocyte development, leading to recurrent opportunistic infections and failure to thrive. Without treatment, most infants succumb in the first year of life.^1^ Causative variants in over 20 genes have been identified to date.^2^ Allogeneic haematopoietic stem cell transplantation (HSCT) has been the standard curative therapy for nearly 60 years, with survival improving from 65% to 70% historically to 85% to 90% in the recent series.^3^, ^4^, ^5^, ^6^, ^7^

The role of conditioning for SCID HSCT has always been contentious. Unconditioned HSCT may be life‐saving in infants with active infections, rapidly providing donor T cells, but often results in poor myeloid and B‐cell engraftment, requiring long‐term immunoglobulin replacement (IgR) leading to suboptimal quality of life.^8^, ^9^ Conditioning improves T‐ and B‐cell reconstitution and long‐term quality of life, but carries risks of chemotherapy‐related toxicity and late effects.^4^, ^5^, ^10^, ^11^ To mitigate these, ESID/EBMT Inborn Errors Working Party guidelines now recommend a reduced toxicity regimen with either pharmacokinetically guided busulfan, or treosulfan irrespective of underlying genotype and donor type.^12^

In the two supra‐regional immunology transplant centres in the United Kingdom, treosulfan replaced busulfan in 2006 for its low toxicity, combined with fludarabine for SCID HSCT. The UK retrospective multicentre treosulfan study has recently demonstrated treosulfan‐fludarabine (TreoFlu) was associated with low rates of endothelial cell dysfunction (ECD) and transplant‐related mortality (TRM) in 537 paediatric patients, of which 42 (7.8%) were infants with SCID.^13^ The present extended study recruited SCID infants who received TreoFlu between 2006 and 2022, focusing on dosing practice and predictors of transplant outcomes.

## METHODS

### Patients

During the study period, 104 SCID infants received TreoFlu for their first allograft at the Great North Children's Hospital (*n* = 72) or Great Ormond Street Hospital (*n* = 32). Written informed consent was obtained from their parents or legal guardians of the patients as per institutional practice for HSCT. The data were retrieved from the transplantation databases, patients' medical files and laboratory records.

### Transplantation

Both study centres used TreoFlu conditioning for SCID during the study period. Treosulfan dosing was based on the institutional practice at the time of transplant, either according to age at HSCT or body surface area (BSA). Total fludarabine dose was 150 mg/m^2^ (*n* = 79), 160 mg/m^2^ (*n* = 18) or 6 mg/kg (*n* = 8). A minority of patients received thiotepa in addition to TreoFlu (*n* = 6). Serotherapy and Graft‐versus‐host disease (GvHD) practice varied according to donor choice and institutional practice.

### Definition and end‐points

Primary outcomes of interest were cumulative incidence (CIN) of ECD, overall survival (OS) and event‐free survival (EFS). OS was survival from first HSCT to last follow‐up or death. EFS was survival without graft failure or second procedures. ECD included patients who developed veno‐occlusive disease (VOD) and/or transplant‐associated microangiopathy (TMA). VOD was diagnosed according to European Society for Blood and Marrow Transplantation (EBMT) diagnostic criteria in children while TMA was diagnosed according to Jodele criteria.^14^, ^15^ Other outcomes assessed were engraftment, GvHD, donor chimerism and IgR dependence at last follow‐up.

### Statistical analysis

Quantitative variables were summarised with median and range while categorical variables were expressed with counts and percentages. Cox Proportional Hazard models assessed factors affecting OS and EFS. The variables assessed in the univariable analyses (UVA) were newborn diagnosis, age at transplant (<3 months vs. ≥3–12 months vs. ≥12 months), SCID genotype (*IL2RG‐JAK3‐IL7R* deficient vs. *RAG‐DCLRE1C* vs. *ADA* vs. other defined genotype vs. undefined/unknown), Omenn features, pre‐HSCT cytomegalovirus (CMV) infection, *Pneumocystis jirovecii pneumonia* (PJP), respiratory syncytial virus (RSV), parainfluenza and gut viral infections, disseminated Bacillus Calmetter‐Guérin (BCG), history of Paediatric Intensive Care unit (PICU) admission, donor (matched family donor [MFD] vs. matched unrelated donor [MUD] vs. mismatched unrelated donor [MMUD] and haploidentical family donor), stem cell source (marrow vs. unmanipulated peripheral blood stem cells [PBSC] vs. T‐cell depleted [TCD] PBSC vs. cord blood [CB]) and treosulfan dose (30 g/m^2^ vs. 36 g/m^2^ vs. 42 g/m^2^). All factors associated with a *p*‐value <0.05 in the UVA for OS and EFS were included in the multivariable analysis (MVA) of the respective outcome. Fine‐and‐Gray competing risk regression was used to estimate the cumulative incidence (CIN) of ECD and GvHD with death and/or as competing events. All *p*‐values quoted are two‐sided, with a level of significance of 0.05. All statistical analyses were performed using STATA 18 (StataCorp LLC, Colleage Station, TX, USA) and graphs were generated with GraphPad Prism version 10.0 (GraphPad Software, LLC, San Diego, California, USA).

## RESULTS

### Study population

Details of patient and transplantation characteristics including treosulfan dosing are summarised in Tables 1 and 2. The median age at diagnosis was 3.5 months (range, birth to 19.8 months). Twenty‐four patients (23%) were diagnosed at birth (21 for a positive family history; 3 via newborn screening). Ninety‐four (90%) patients had genetically defined SCID, 7 (8%) were undefined despite whole‐genome sequence (WGS) and 3 (2%) were unknown (Tables S1 and S2). The median age at HSCT was 6.9 months (2.1–28.8 months) and the median interval between diagnosis and HSCT was 2.8 months (0.5–23.6 months). The median age at HSCT was 2.1 months (1.4–14.7 months) in newborn‐diagnosed SCID and 8.0 months (2.3–43.3 months) in symptomatic patients (*p* < 0.001). There was no significant difference in the interval between diagnosis and HSCT between the two groups (newborn‐diagnosed SCID: median 2.1 months [range 1.4–13.3] vs. symptomatic: median 3.1 months [range 0.5–23.1]; *p* = 0.19). Three patients (ADA, *n* = 1; IL2RG, *n* = 2) had gene therapy prior to transplant.

**TABLE 1:** Patient and transplant characteristics.

SCID genotype, *n* (%)	
IL2RG	22 (23)
JAK3	8 (8)
IL7R	10 (10)
ADA	12 (12)
RAG1/2	27 (28)
DCLRE1C	10 (10)
AK2	1 (1)
CD3 delta	2 (2)
CD3 epsilon	1 (1)
LAT	1 (1)
Undefined despite WGS	7 (8)
Unknown (WGS not done)	3 (2)
Pretransplant issues, *n* (%)	
Omenn features	31 (30)
PJP	35 (34)
Disseminated BCG	14 (13)
CMV viraemia	12 (12)
Adenoviraemia	7 (7)
Gut viruses	42 (40)
Number of patients with ≥2 viruses	13 (13)
Type of viruses	
Rotavirus	21 (20)
Norovirus	19 (18)
Adenovirus	2 (2)
Enterovirus	4 (4)
Sapovirus	2 (2)
Astrovirus	1 (1)
Respiratory viruses	40 (38)
Number of patients with ≥2 viruses	13 (13)
Type of viruses	
RSV	7 (7)
Parainfluenza	10 (10)
Rhinovirus	26 (25)
Metapneumovirus	2 (2)
Adenovirus	2 (2)
Bocavirus	2 (2)
Enterovirus	2 (2)
Influenzae A/B	2 (2)
Seasonal coronavirus	2 (2)
Parechovirus	1 (1)
PN dependent	25 (24)
History of PICU admission	18 (17)
Number of patients received gene therapy prior to transplant	3 (1 ADA, 2 IL2RG)
Donor characteristics	
Donor type, *n* (%)	
Matched family donor	32 (30)
Matched unrelated donor	33 (31)
Mismatched unrelated donor	21 (20) [17 cord; 2 marrow; 1 PBSC; 1 T‐cell depleted PBSC]
Haploidentical family donor (≤8/10)	19 (18)
Stem cell source, *n* (%)	
Marrow	26 (25)
Unmanipulated PBSC	26 (25)
T‐cell depleted PBSC^a^	20 (19)
Cord blood	32 (31)
Graft details	
Median TNC (range), ×10^8^/kg	7.9 (0.1–44.5)
Median CD34+ (range), ×10^6^/kg	7.4 (0.05–60.9)
Median CD3+ (range), ×10^8^/kg	0.59 (0–10.0)
Transplant characteristics	
Conditioning	
Treosulfan–fludarabine, *n* (%)	98 (94)
Fludarabine–treosulfan–thiotepa, *n* (%)	6 (6)
Serotherapy	
None, *n* (%)	15 (14)^b^
ATG, *n* (%)	25 (24)
Alemtuzumab, *n* (%)	64 (62)
GVHD prophylaxis	
None, *n* (%)	10 (10)
CSA alone, *n* (%)	4 (4)
CSA + MMF, *n* (%)	88 (84)
CSA + steroids, *n* (%)	2 (2)

*Note*: +One ADA patient with failed gene therapy was transplanted at 43.3 months.

Abbreviations: ATG, anti‐thymocyte globulin; CMV, cytomegalovirus; CSA, ciclosporin; GvHD, graft‐versus‐host disease; MMF, mycophenolate mofetil; PBSC, peripheral blood stem cells; PJP, Pneumocystis Jirovecii pneumonia; RSV, respiratory syncytial virus; SCID, severe combined immunodeficiency; TNC, Total nucleated cell dose; WGS, whole‐genome sequence.

^a^ TCRαβ/CD19 depleted PBSC: 17; CD3/CD19 depleted PBSC: 3.

^b^ MFD marrow (*n* = 3), CB (*n* = 11; HLA matching: 10/10, *n* = 2; 9/10, *n* = 4; ≤8/10, *n* = 5), CD3/CD19 depleted PBSC (*n* = 1).

**TABLE 2:** Treosulfan dosing according to body surface area (BSA)and age group at transplant.

	*n*	Treosulfan dose, *n*		
		30 g/m^2^	36 g/m^2^	42 g/m^2^
Body surface area				
<0.5	103	34	55	14
≥0.5 to <1.0	1^a^	0	0	1
Age group				
<3 months	22	14	8	0
≥3 and <12 months	70	15	49	6
≥12 months	15	4	1	10

^a^ This patient was transplanted at 43.3 months after failed gene therapy.

### Clinical outcome

All achieved neutrophil engraftment (median 17 days; range 9–46 days) and platelet engraftment (median 19 days; range 7–79 days) except one who died prior to platelet engraftment.

**FIGURE 1:**
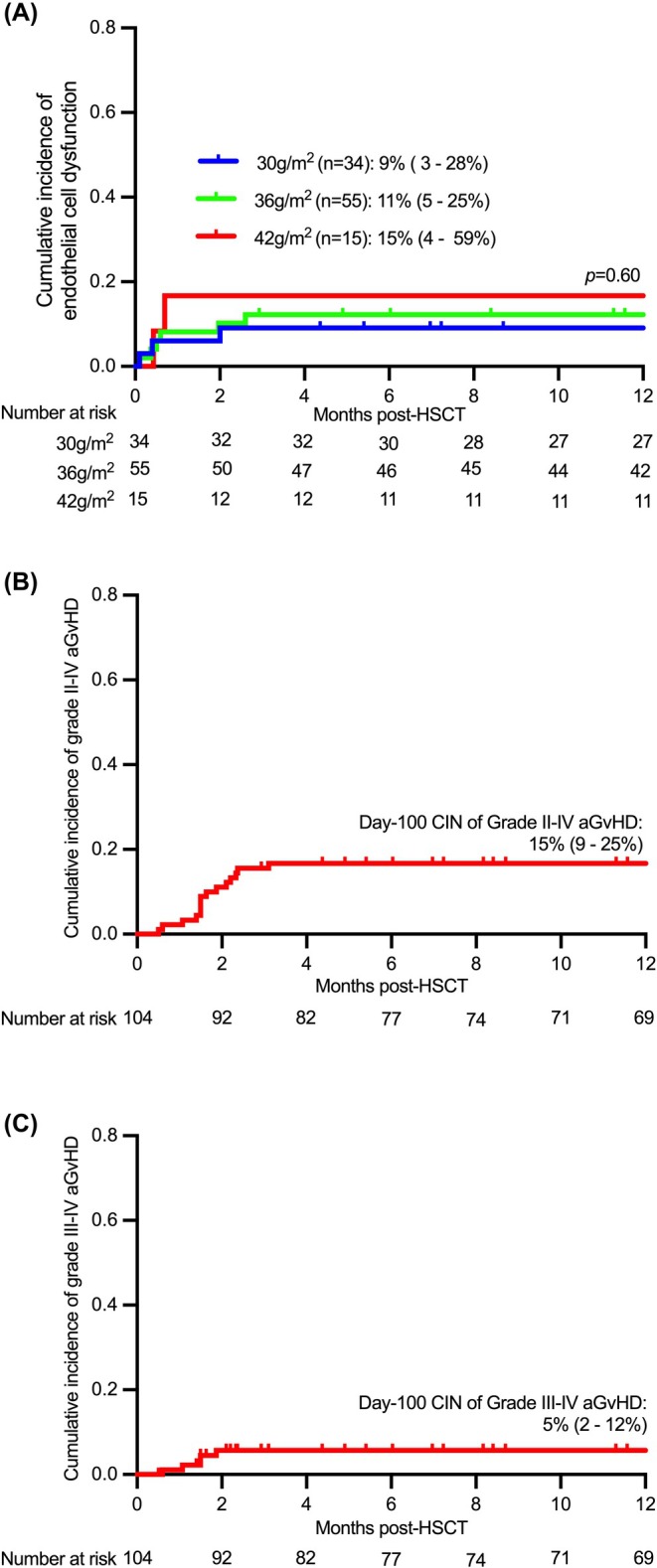
(A) Cumulative incidence of endothelial cell dysfunction, stratified by treosulfan dose. Cumulative incidence of grade II–IV acute GvHD (B) and grade III–IV acute GvHD (C).

The day‐180 CIN of ECD was 11% (95% confidence interval (CI), 6%–20%) for the entire cohort, 9% (3%–28%) after Treo 30 g/m^2^, 11% (5%–25%) after Treo 36 g/m^2^, 14.8% (4%–59%) after Treo 42 g/m^2^ (*p* = 0.60) (Figure 1A). Of 6 (6%) patients with VOD, treosulfan dose was 36 g/m^2^ in four, 42 g/m^2^ in two and none had thiotepa. Six (6%) developed TMA; treosulfan dose was 30 g/m^2^ in three patients (2 undefined SCID, 1 *JAK3*), 36 g/m^2^ in two (1 *DCLRE1C*, 1 *IL2RG*), 42 g/m^2^ in one (*DCLRE1C*) and none received thiotepa.

Four patients had persistent CMV viraemia from pretransplant; 10 (10%) had new onset CMV viraemia after HSCT. Nine had post‐transplant adenoviraemia (8.7%), 3 (2.8%) had HHV6 viraemia and 4 (3.8%) had Epstein‐Barr virus (EBV) viraemia (one of whom received rituximab; none had post‐transplant lymphoproliferative disease).

Fifty‐six patients (53.8%) received parental nutrition during transplant; 22 (39%) were started pretransplant for viral enteropathy, 2 (4%) for gut rest (institutional practice for CB transplant) and 32 (57%) for mucositis post‐transplant.

The day‐100 CIN of grade II–IV aGvHD and III–IV was 15% (9%–25%) and 5.0% (2%–12%) respectively (Figure 1B,C). Of five patients with grade III–IV aGvHD, two received matched CB (both received alemtuzumab), two received mismatched CB (1 T‐depleted with ATG; 1 without serotherapy) and one received unmanipulated PBSC. Two resolved with treatment, one died of GvHD (mismatched CB without serotherapy) and two patients received a second successful transplant for refractory GvHD. None had chronic GvHD. Four (3.8%) developed immune cytopenia post‐transplant; two developed isolated immune haemolytic anaemia and two developed combined immune haemolytic anaemia and thrombocytopenia. All received immunomodulators (2 received 2 therapies; 2 received ≥3 therapies) and one died of infection.

Two (1.9%) developed secondary leukaemia; both had RAG deficiency with complete loss of donor myeloid chimerism and cytogenetic analysis confirmed that host‐derived leukaemia (opposite‐sex donor). One died during chemotherapy; the other one underwent a successful second transplant.

Early mortality (≤1 year post‐HSCT) occurred in 12 patients, due to infection (*n* = 4; CMV, 3; influenza A, 1), pneumonitis (*n* = 4), thrombotic microangiopathy (*n* = 2), GvHD (*n* = 1) and immune reconstitution syndrome (*n* = 1). Among five late deaths, the median time from HSCT to death was 2.5 years (2.06–3.99), with causes including infection (*n* = 2; both on prolonged immunosuppression for GvHD and autoimmune cytopenia), respiratory failure (*n* = 2) and leukaemia (*n* = 1).

### Impact of SCID genotype on OS and EFS

**FIGURE 2:**
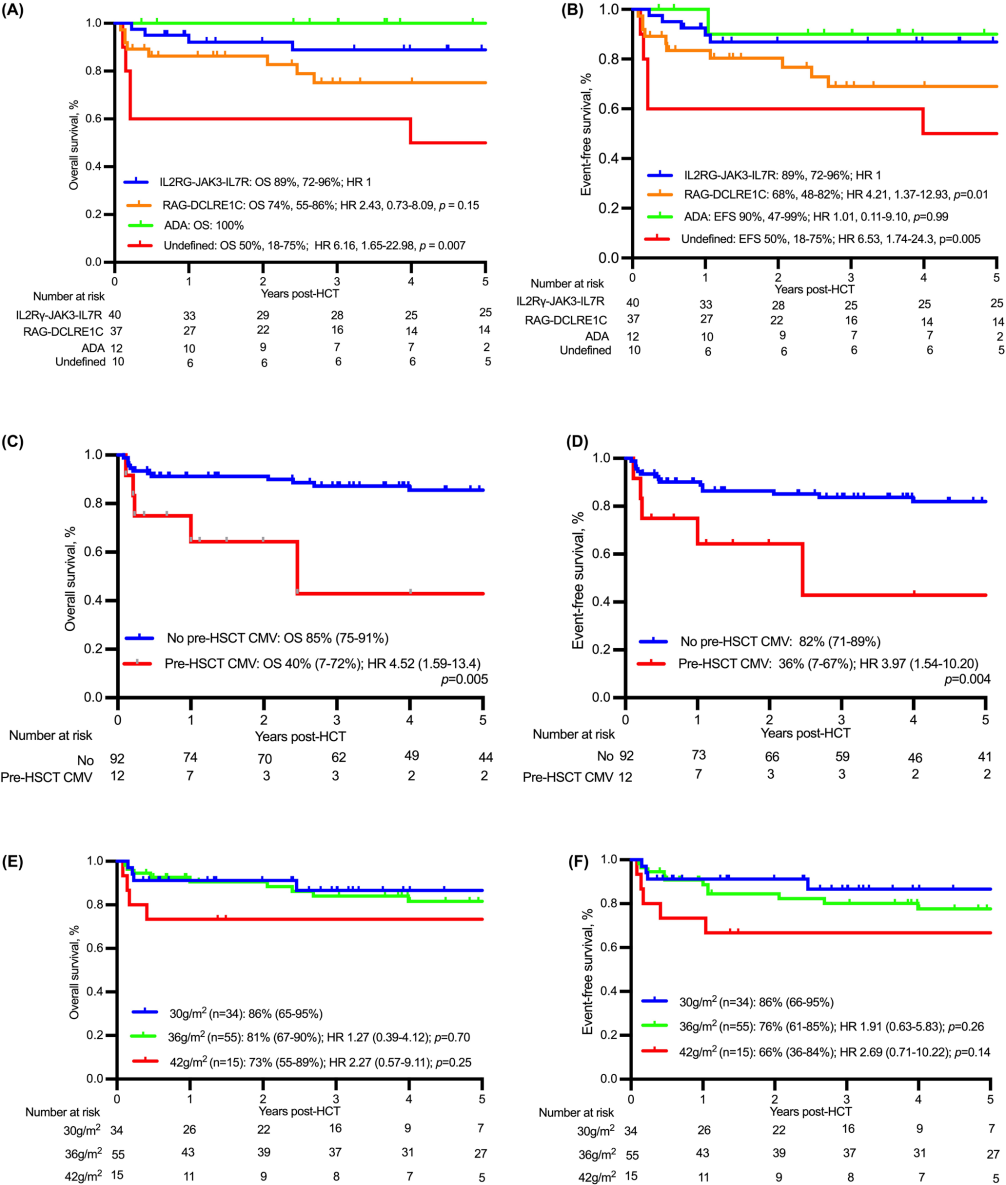
Overall survival (OS) (A) and event‐free survival (EFS) (B), stratified by genotype. OS (C) and EFS (D) by pretransplant cytomegalovirus (CMV) infection. OS (E) and EFS (F) by treosulfan dose.

The median follow‐up of surviving patients was 5.4 years (0.24–15.3 years). The 1‐year and 5‐year OS for the entire cohort was 88% (79%–93%) and 81% (71–88%) respectively. Compared to a 5‐year OS of 89% (72%–96%) for the group of *IL2RG‐JAK3‐IL7R‐*deficient (T‐B+; *n* = 40) SCID patients, the OS was 74% (55%–86%) for *RAG‐DCLRE1C*‐deficient patients (T‐B‐ SCID; *n* = 37, hazard ratio [HR] 2.43, 0.73–8.09, *p* = 0.15) and 50% (18–75%) for undefined/unknown genotype patients (*n* = 10; HR 6.16, 95% CI 1.65–22.98, *p* = 0.007, Table 3; Figure 2A). OS was 100% for ADA (*n* = 12) and other genotypes (*n* = 5; AK2 defect, *n* = 2; CD3 delta, *n* = 2, CD3 epsilon, *n* = 1 and LAT, *n* = 1).

**TABLE 3:** Univariate analysis for OS and EFS.

	*n*	OS			EFS		
		5‐year OS (95% CI)	HR (95% CI)	*p‐*value	5‐year EFS (95% CI)	HR (95% CI)	*p*‐value
Age							
<3 months	22	84 (58–95)	1		84 (58–95)	1	
≥3 to <12 months	67	82 (69–89)	1.29 (0.36–4.63)	0.69	77 (64–86)	1.95 (0.57–6.70)	0.29
≥12 months	25	78 (46–92)	1.88 (0.38–9.34)	0.44	68 (35–87)	2.68 (0.60–12.0)	0.20
Newborn diagnosis							
No	75	79 (66–88)	1		73 (61–83)	1	
Yes	29	88 (67–96)	0.53 (0.15–1.84)	0.32	88 (67–96)	0.50 (0.17–1.48)	0.21
SCID genotype							
IL2RG‐JAK3‐IL7R	40	89 (72–96)	1		89 (72–96)	1	
RAG‐DCLRE1C	37	74 (55–86)	2.43 (0.73–8.09)	0.15	68 (48–82)	4.21 (1.37–12.93)	0.01
ADA	12	100	—	—	90 (48–82)	1.01 (0.11–9.10)	0.99
Minor genotype	5	100	—	—	100	—	—
Undefined/unknown	10	50 (18–75)	6.16 (1.65–22.98)	0.007	56 (20–80)	6.53 (1.75–24.38)	0.005
Omenn features							
No	73	84 (72–91)	1		83 (71–88)	1	
Yes	31	75 (54–87)	1.84 (0.79–4.84)	0.22	64 (43–79)	2.45 (1.08–5.57)	0.032
Pre‐HSCT CMV infection							
No	92	85 (75–91)	1		82 (71–89)	1	
Yes	12	40 (7–72)	4.62 (1.59–13.4)	0.005	36 (7–67)	3.97 (1.54–10.2)	0.004
Pre‐HSCT PJP							
No	69	80 (67–88)	1		78 (65–87)	1	
Yes	35	83 (65–93)	0.77 (0.27–2.18)	0.62	75 (56–87)	1.00 (0.42–2.37)	0.99
Pre‐transplant RSV							
No	97	81 (71–88)	1		76 (66–84)	1	
Yes	7	86 (33–98)	0.86 (0.11–6.51)	0.89	86 (33–98)	0.58 (0.08–4.32)	0.60
Pre‐HSCT Parainfluenza							
No	94	80 (70–88)	1		73 (65–84)	1	
Yes	10	90 (47–99)	0.54 (0.07–4.06)	0.55	90 (47–99)	0.38 (0.05–2.84)	0.35
Pre‐HSCT gut viruses							
No	62	88 (77–95)	1		84 (71–91)	1	
Yes	42	70 (52–82)	2.85 (1.05–7.70)	0.04	61 (41–78)	2.10 (0.92–4.79)	0.08
Pre‐HSCT disseminated BCG							
No	87	80 (69–88)	1		78 (65–85)	1	
Yes	18	86 (55–96)	0.66 (0.15–2.89)	0.58	79 (47–93)	0.74 (0.2–2.48)	0.63
History of PICU admission							
No	86	80 (68–86)	1		73 (61–82)	1	
Yes	18	94 (66–99)	0.26 (0.03–1.98)	0.20	94 (38–96)	0.39 (0.09–1.67)	0.20
By donor type							
MFD	32	75 (54–87)	1		75 (55–87)	1	
MUD	32	88 (72–97)	0.42 (0.11–1.62)	0.21	78 (56–88)	0.91 (0.33–2.52)	0.86
MMUD	21	75 (50–89)	0.99 (0.32–3.13)	0.99	75 (50–89)	0.82 (0.27–2.52)	0.74
Haploidentical family donor	19	88 (58–98)	0.35 (0.10–2.29)	0.35	88 (58–97)	0.64 (0.17–2.43)	0.52
Stem cell source							
Marrow	26	83 (60–93)	1		79 (57–91)	1	
Unmanipulated PBSC	26	78 (55–90)	1.60 (0.43–6.01)	0.48	78 (54–90)	1.15 (0.39–3.78)	0.82
T‐cell depleted PBSC*	20	83 (55–94)	1.07 (0.24–4.80)	0.93	83 (55–94)	1.01 (0.28–3.58)	0.99
CB	32	84 (65–93)	0.97 (0.26–3.61)	0.96	74 (54–87)	1.05 (0.36–3.03)	0.93
By Treo dose							
30 g/m^2^	34	86 (66–95)	1		86 (66–95)	1	
36 g/m^2^	55	81 (67–90)	1.27 (0.39–4.12)	0.70	76 (61–85)	1.91 (0.63–5.83)	0.26
42 g/m^2^	15	73 (55–89)	2.35 (0.59–9.41)	0.23	66 (36–84)	2.69 (0.71–10.22)	0.14

Abbreviations: CB, cord blood; EFS, event‐free survival; HR, hazard ratio; MFD, matched family donor; MMUD, mismatched unrelated donor; MUD, matched unrelated donor; OS, overall survival; PBSC, peripheral blood stem cells; RSV, respiratory syncytial virus.

* CD3/19 depletetion, *n* = 3; TCRαβ/CD19 depletion, *n* = 17.

The 1‐year and 5‐year EFS for the entire cohort was 82% (73%–87%) and 77% (67%–85%). Six patients received a successful second conditioned transplant (2 refractory aGvHD, 2 poor immune reconstitution, 1 secondary leukaemia and 1 absent donor myeloid with chronic aichi viral hepatitis). Of two patients who received donor lymphocyte infusion (DLI) for poor immune reconstitution, one died of inflammatory pneumonitis after DLI and one was alive at 15 years post‐transplant. Compared to IL2RG‐JAK3‐IL7R‐deficient patients (89%, 73%–96%), RAG‐DCLRE1C‐deficiency (68%, 48%–82%; HR 4.21, 1.37–12.93, *p* = 0.01) and undefined/unknown genotype (56%, 20%–80%; HR 6.53, 1.75–34.38, *p* = 0.005) were associated with unfavourable EFS. EFS was 90% (47%–99%, *p* = 0.81) for ADA and 100% for other genotype groups (Figure 2B).

### Impact of patient factors and pre‐transplant infection on OS and EFS

Compared to infants <3 months, there was no significant difference in OS and EFS in infants aged between 3 and 12 months and >12 months (Table 3). Newborn diagnosis had no significant association with OS (*p* = 0.32) and EFS (*p* = 0.21).

While the presence of Omenn features (*n* = 31) had no association with OS (75%, 54%–87% vs. absence 84%, 72%–91%, *p* = 0.22), EFS was inferior in patients with Omenn features (64%, 43%–79% vs. absence 83%, 71%–88%, *p* = 0.03). The genotypes of 31 patients with Omenn features were RAG1/RAG2 deficiency (*n* = 16, 52%), DCLRE1C (*n* = 4, 12%), JAK3 (*n* = 4, 12%), IL7R (*n* = 3, 10%), undefined (*n* = 4, 12%) and unknown (*n* = 1, 2%).

Pre‐transplant CMV infection was associated with inferior OS (*p* = 0.005) and EFS (*p* = 0.004) compared to patients without CMV infection (Figure 2C,D). Unfavourable OS was observed in patients with gut viruses (*p* = 0.04) compared to patients without gut viruses, but the association was not significant in EFS (*p* = 0.08). PJP, RSV, parainfluenza and disseminated BCG had no significant impact on OS or EFS. History of PICU admission pre‐HSCT did not affect OS (*p* = 0.20) or EFS (*p* = 0.20) in patients who were stabilised prior to conditioned HSCT.

### Impact of donor and stem cell source on OS and EFS

As summarised in Table 3, OS and EFS were comparable between MFD (*n* = 32), MUD (*n* = 32), MMUD (*n* = 21 including 17 CB) and haploidentical family donor (*n* = 19). OS and EFS were also comparable between marrow (*n* = 25), unmanipulated PBSC (*n* = 26), T‐cell depleted PBSC (*n* = 20; 17 TCRαβ/CD19 depletion, 3 CD3/CD19 depletion) and CB (*n* = 32). Human leukocyte antigen (HLA) ‐matching for CB was 10/10 in 15 (47%) recipients, 9/10 in 9 (28%) and ≤8/10 in 8 (25%).

### Impact of treosulfan dose on OS and EFS

All patients except one had a body surface area (BSA) <0.5 m^2^ at HSCT. One patient who was transplanted at 43.3 months after failed gene therapy had a BSA of 0.62 m^2^. Treosulfan dose according to BSA and age group are summarised in Table 2. All received fludarabine and treosulfan and six patients received additional thiotepa (2 IL2RG; 1 RAG1; 1 IL7Ra; 1 reticular dysgenesis; 1 ADA).

The 5‐year OS was 86% (66%–95%) for treosulfan 30 g/m^2^ (*n* = 34), 81% (67%–90%; HR 1.27, 0.39–4.12, *p* = 0.70) for 36 g/m^2^ (*n* = 55) and 73% (55%–89%; HR 2.27, 0.57–9.11, *p* = 0.25) for 42 g/m^2^ (*n* = 14) (Figure 2E). EFS was 86% (66%–95%) for treosulfan 30 g/m^2^ and 76% (61%–85%) for 36 g/m^2^ (HR 1.91, 0.63–5.83, *p* = 0.26) and 66% (36%–84%) for 42 g/m^2^ (HR 2.69, 0.71–10.22, *p* = 0.14, Figure 2F).

### Multivariable analysis for OS and EFS

In MVA for OS and EFS, two independent factors were significantly associated with unfavourable OS and EFS: undefined/unknown genotype (HR 5.61, 1.45–21.60, *p* = 0.01; HR 5.55, 1.50–20.56, *p* = 0.01 respectively) and pretransplant CMV infection (HR 3.94, 1.32–11.75, *p* = 0.01; HR 3.68, 1.36–9.90, *p* = 0.01) (Table 4). EFS was also significantly inferior in RAG‐DCLRE1C‐deficient patients (HR 4.35, 1.35–14.10, *p* = 0.01). Treosulfan dosing for RAG‐DCLRE1C‐deficient patients is summarised in Table S3, with comparable dose distribution between both genotypes. At 30 g/m^2^, EFS was 87.5% for RAG (*n* = 8) and 100% (*n* = 3) for DCLRE1C; at 36 g/m^2^, 66.7% (*n* = 15) and 40% (*n* = 5); at 42 g/m^2^, 40% (*n* = 4) and 50% (*n* = 2).

**TABLE 4:** Multivariate analysis for OS and EFS.

	OS		EFS	
	HR (95% CI)	*p*‐value	HR (95% CI)	*p*‐value
SCID genotype				
RAG‐DCLRE1C (1)	2.16 (0.64–7.27)	0.21	4.35 (1.35–14.10)	0.01
Undefined/unknown	5.61 (1.45–21.6)	0.01	7.43 (1.89–29.26)	0.004
Pretransplant CMV infection	3.94 (1.32–11.75)	0.01	3.18 (1.12–9.07)	0.03
Pretransplant gut virus	1.84 (0.66–5.12)	0.24	—	—
Omenn features	—	—	0.88 (036–2.18)	0.79

Abbreviations: CMV, cytomegalovirus; EFS, event‐free survival; HR: hazard ratio; OS, overall survival; SCID, severe combined immunodeficiency.

### Donor chimerism and immunoglobulin replacement dependence at latest follow‐up

**FIGURE 3:**
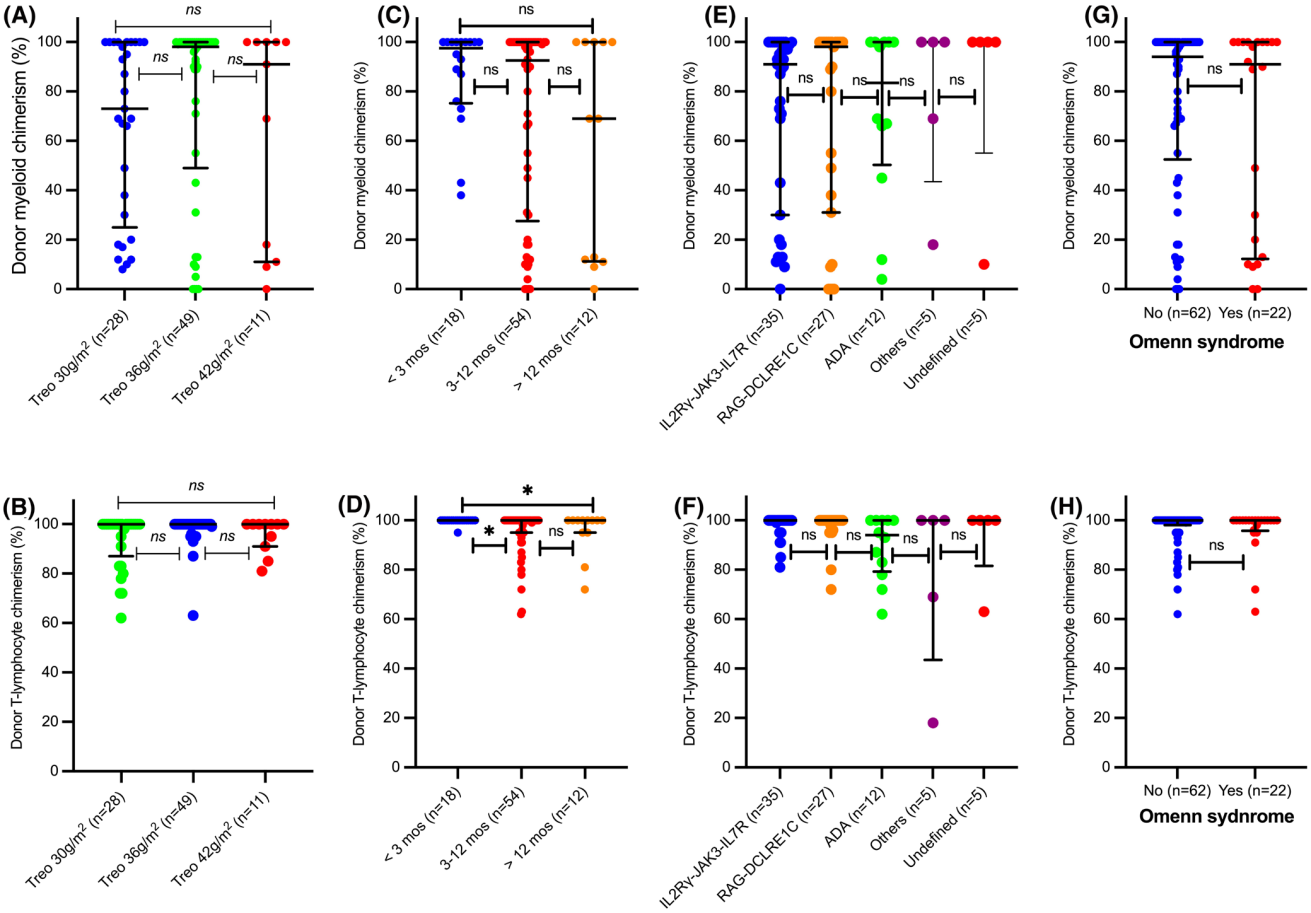
Donor myeloid and T‐lymphocyte chimerism by treosulfan dose (A, B), age at haematopoietic stem cell transplantation (HSCT) (C, D), genotype (E, F) and presence of Omenn syndrome (G, H).

Of 87 survivors, long‐term data were available in 83 with four patients lost to follow up. The median follow‐up of these 83 patients was 8.3 years (1.28–17.9 years). Analysis of donor chimerism at last follow‐up (including pre‐second HSCT in the 6 patients who received second HSCT) demonstrated no significant association between treosulfan dose and the degree of donor myeloid and T‐lymphocyte chimerism (Figure 3A,B). Low mixed myeloid chimerism (<20%) was observed across all treosulfan doses: 6 of 28 patients (21%) at 30 g/m^2^ (ADA, 3; IL7R, 1; RAG1, 1; CD3δ), 9 of 42 (21%) at 36 g/m^2^ (RAG1/RAG2, 5; JAK3, 2; IL2RG, 1; undefined, 1) and 4 of 11 (36%) at 42 g/m^2^ (RAG1, 1; IL7R, 2; IL2RG, 1). Age <3 months was associated with higher T‐lymphocyte chimerism compared with 3–12 months (*p* = 0.02) and >12 months (*p* = 0.04); however, this association was not observed for myeloid chimerism (Figure 3C,D). There was no significant difference in donor myeloid and T‐lymphocyte chimerism according to SCID genotype (Figure 3E,F) and the presence of Omenn syndrome (Figure 3G,H).

Of 76 survivors after first HSCT, 70 (92%) achieved independence from immunoglobulin replacement (IgR). Among the six who remained IgR dependent, the median follow‐up was 3.87 years (2.12–7.39 years), with a median myeloid chimerism of 13% (0%–73%). Of six patients who underwent second HSCT, all had full donor chimerism; four were IgR independent, one remained IgR dependent and one was only 6 months post second HSCT at the time of analysis.

## DISCUSSION

This is the largest retrospective study to date evaluating outcomes in patients with SCID who underwent HSCT using a treosulfan‐fludarabine conditioning regimen. Our data confirm that this combination is safe and effective with outcomes comparable to other large SCID HSCT transplant series.^6^, ^16^ Treosulfan exhibits a more favourable endothelial toxicity profile compared to busulfan, although there is no study directly comparing treosulfan and busulfan in SCID transplant. No significant differences in OS or EFS were observed according to treosulfan dose, donor type or stem cell sources. However, an undefined genotype and the presence of CMV infection before transplantation were associated with inferior OS and EFS on MVA. RAG‐DCLRE1C‐deficient patients also showed poorer EFS. Mismatched unrelated and haploidentical donors did as well as matched donors in this report in contrast to the large European series which reported an inferior 2‐year OS using mismatched donors.^16^ More than 90% patients in this report were able to stop immunoglobulin replacement confirming the efficacy of conditioning in achieving B‐cell engraftment.

The prognostic importance of a known molecular diagnosis has been highlighted in previous reports. Forlanini et al. reported 98 patients with primary immunodeficiencies (PID) transplanted between 2007 and 2018, including 43 with SCID. Thirty‐five patients (43%) had a confirmed genotype at HSCT, and their 5‐year OS was 94% compared with 56% in those without a genetic diagnosis.^17^ The possible explanations for poorer outcomes in genetically undefined SCID include delayed diagnosis, thymic defects masquerading as SCID, uncertainty regarding optimal conditioning or underlying conditions that could have made HSCT more toxic. In undefined SCID, artificial thymic organoids should be performed as it provides a reliable platform to study T‐cell differentiation and help distinguish between haematopoietic‐intrinsic and extra‐haematopoietic defects causing T‐cell lymphopenia.^18^

Pretransplant infections have long been recognised as a major adverse prognostic factor and were the key driver behind the introduction of newborn screening around the world.^6^, ^16^ While we found no difference in the outcome for the 24 patients identified with SCID at birth compared to the rest due to small number in our study, Thakar et al. reported a significant improvement in OS when patients were transplanted at a younger age without infections, both directly driven by newborn screening.^19^

EFS was significantly unfavourable in RAG‐DCLRE1C‐deficient patients. It is well known that these patients require myeloablation to achieve durable immune reconstitution due to residual natural killer (NK)‐cell function and in order to empty marrow and thymic niches that inhibit donor progenitor engraftment, but this needs to be balanced with the increased late effects seen in association with the use of myeloablative chemotherapeutic agents in infants with RAG‐SCID, particularly DCLRE1C‐SCID because of systemic radiosensitivity.^4^, ^20^

With the advent of newborn screening and the excellent outcome in terms of survival from HSCT in the modern era, it is imperative to optimise the long‐term quality of life for these patients. This includes good immune reconstitution and freedom from late effects. Alkylating agents impair gonadal function and fertility, and myeloablative busulfan regimens cause severe gonadotoxicity, particularly in females. Emerging evidence shows that treosulfan is less gonadotoxic than busulfan.^21^, ^22^ Van der Stoep et al. reported 57 patients with non‐malignant diseases who were conditioned with either busulfan‐ or treosulfan‐based conditioning. In the busulfan group, 56 patients could be evaluated, and gonadal dysfunction was found in 35 (63%). Lower busulfan exposure (50–70 mg × h/L) was not associated with a lower risk of gonadal dysfunction. In the treosulfan cohort, 32 patients were evaluable and gonadal insufficiency occurred in nine patients (28%).^23^

Dosing of treosulfan varies between centres around the world according to age, weight and body surface (BSA).^24^, ^25^ Dosing of treosulfan in this report was variable (Table 2) and there was no association with dose and subsequent chimerism. Medac BSA recommended dosing at the time was 30 g/m^2^ for patients <0.5 m^2^ and has recently revised the BSA dosing recommendations to 10 g/m^2^, 12 g/m^2^ and 14 g/m^2^ for BSAs of <0.4 m^2^, ≥0.4 to <0.9 m^2^ and ≥0.9 m^2^ respectively.^26^ Pharmacokinetic (pK) studies are emerging but therapeutic drug monitoring‐based dosing is not routinely available. We have previously reported an association with high area under the curve (AUC) for treosulfan and mortality, and low myeloid chimerism of <20% with a low AUC for treosulfan in 87 children (median age of 19 months), 79 of whom had a variety of primary immunodeficiencies.^27^ A number of other pK studies of treosulfan in children have been performed and all recommend lower dosing in infants. BSA‐based dosing should give similar AUC for children older than around 2 years, but it is well known that, in the first year of life, BSA‐based dosing leads to higher AUC due to immaturity in clearance.^28^ As expected in this current report of purely SCID patients, the median age at HSCT was much lower at nearly 7 months, and therefore, this represents a unique cohort of infants. Twelve patients who received only fludarabine and treosulfan as conditioning had endothelial cell dysfunction, which may be related to toxicity of chemotherapy.

There is a growing interest in studying fludarabine pK in HSCT and serotherapy type and timing are recognised as major determinants of GVHD and immune reconstitution.^29^, ^30^, ^31^, ^32^, ^33^, ^34^ Therefore, a personalised approach to conditioning a patient with SCID should ideally include pharmacokinetic monitoring of all agents contained in the conditioning regimen in order to optimise engraftment and robust immune reconstitution and minimise short‐ and long‐term adverse effects.

## AUTHOR CONTRIBUTIONS

S.H.L conceptualised the research, collected the data, performed the statistical analysis, interpreted the data and prepared the manuscript. S.G. collected the data, interpreted the data and prepared the manuscript. M.S. and K.R. contributed in conceptualising the research, collected the data and prepared the manuscript. I.L.M., P.A, Z.N., R.C., J.S., H.Y., S.O., E.W., K.F.N., T.F., A.G. and S.H. contributed the data and/or reviewed the manuscript.

## FUNDING INFORMATION

The authors received no financial support for the research, authorship and/or publication of this article.

## CONFLICT OF INTEREST STATEMENT

The authors declare that there is no conflict of interest.

## PATIENT CONSENT STATEMENT

Patients and/or their parents/legal guardians were consented for inclusion of their anonymous data for audit of clinical outcome at the time of transplant consent according to institutional guideline.

## Supporting information


Table S1.
Table S2.
Table S3.

## Data Availability

The data that support the findings of this study are available from the corresponding author.
