# Supplementary material for: Treosulfan–fludarabine conditioning in infants with severe combined immunodeficiencies: Extended study of the UK paediatric treosulfan study
Source: Br J Haematol. 2026 Apr 10;208(5):1713–23. doi: 10.1111/bjh.70453 (PMC13176534; doi:10.1111/bjh.70453)
Supplement: Supplementary file 1 — Table S1. Table S2. Table S3. [file BJH-208-1713-s001.docx]

**Supplemental table 1: Clinical features of undefined SCID despite whole exome sequencing**

| **Number** | **Phenotype** | | | |  |  |  |  |  |  |  |
| --- | --- | --- | --- | --- | --- | --- | --- | --- | --- | --- | --- |
|  | **T cells**  (cells/µL) | **B cells**  (cells/µL) | **NK cells**  (cells/µL) | **Naïve T cells**  (cells/µL) | **Age at presentation**  **(months)** | **Age at diagnosis**  **(months)** | **Clinical features** | **Age at transplant**  **(months)** | **Transplant details** | **Transplant complications** | **Outcomes** |
|  | 135 | 4 | 3 | 0 | At birth | 0.9 | Diarrhoea from birth, disseminated adenoviral infection (blood and stools), PN dependent enteropathy | 2.9 | A-MM CB  Flu-Treo36  No serotherapy  CSA/MMF | Grade 2 aGvHD | Died at 4 years of age due to recurrent infection secondary to prolonged immunosuppression for immune dysregulation |
|  | 100 | 548 | 83 | 0 | 1.0 | 3.8 | Recurrent respiratory tract infection, chronic diarrhoea, failure to thrive,  Rotavirus, disseminated CMV (encephalitis, hepatitis, colitis), PDA | 6 | MFD marrow  Flu-Treo36  Alemtuzumab  CSA/MMF | Admitted to PICU on the day of transplant for respiratory and renal failure necessitating renal replacement therapy | Died at day +39 due to multi-organ failure |
|  | 10059 | 2703 | 1316 | NA | 6.0 | 8.8 | Omenn features (rash, lymphadenopathy, hepatosplenomegaly, eosinophilia, raised IgE) | 10.2 | MSD marrow  Flu-Treo36  Alemtuzumab  CSA/MMF | No | Alive  IRT-free  Chimerism  CD15+ 10%  CD3+ 63 |
|  | 403 | 2106 | 232 | 0 | 6.0 | 6.5 | PCP pneumonia complicated by pulmonary haemorrhage, rotavirus enteropathy | 9.5 | Haplo  Flu-Treo  ATG Grafalon  No GvHD prophylaxis  Bellicum addback | Grade 3 aGvHD | Alive  IRT  100% donor chimerism |
|  | 0 | 0 | 199 | 0 | Newborn | Newborn | Omenn’s feature (rash and eosinophilia), positive enterovirus in stools | 2.1 | MFD PBSC  Flu-Treo36  Alemtuzumab  CSA/MMF | Developed capillary leakage, TMA and renal failure and pneumonitis early post-transplant | Died at day +56 due to TMA and pneumonitis |
|  | 0 | 310 | 0 | 0 | 3.5 | 3.5 | Prematurity 33weeks (BW 1.25kg), PCP pneumonia, PN dependent viral enteropathy (norovirus, enterovirus) | 9.5 | MUD PBSC  Flu-Treo30  Alemtuzumab  CSA/MMF | Developed capillary leakage, TMA and pneumonitis early post-transplant | Died at day +75 due to TMA and pneumonitis |
|  | 230 | 630 | 270 | 0 | .0.5 | 8.5 | FTT, PCP pneumonitis | 11.9 | 7/10 MMUD cord  Flu-Treo36  Alemtuzumab  CSA/MMF | Grade 2 aGvHD | Alive  100% WB chimerism |

**Supplemental table 2: Clinical features of SCID patients with no molecular diagnosis (no whole exome sequencing)**

| **Number** | **Phenotype** | | | |  |  |  |  |  |  |  |
| --- | --- | --- | --- | --- | --- | --- | --- | --- | --- | --- | --- |
|  | **T cells**  (cells/µL) | **B cells**  (cells/µL) | **NK cells**  (cells/µL) | **Naïve T cells**  (cells/µL) | **Age at presentation**  **(months)** | **Age at diagnosis**  **(months)** | **Clinical features** | **Age at transplant**  **(months)** | **Transplant details** | **Transplant complications** | **Outcomes** |
| 1. | 6430 | 200 | 1370 | 10 | 0.2 | 0.9 | Skin peeling, FTT, Lymphadenopathy and cough | 2.9 | 7/10 MMUD CB  Flu-Treo36  No serotherapy  CSA/MMF | Grade 4 aGVHD | Died at day +77 due to pulmonary haemorrhage secondary to lung GvHD |
| 2. | 150 | 129 | 210 | 30 | 3.8 | 3.8 | Investigated as identical twin diagnosed as SCID.  Extensive skin rash on legs | 6 | 7/10 MMUD CB  Flu-Treo42  No serotherapy  CSA/MMF | Grade 2 aGvHD | Alive  IRT-free  100% WB chimerism |
| 3. | 130 | 0 | 170 | 13 | 3.8 | 5.3 | Poor feeding, weight loss,  diarrhoea, respiratory tract infection | 7.5 | 8/10 MMUD CB  Flu-Treo36  No serotherapy  CSA/MMF | Grade 1 aGvHD | Alive  IRT-free  100% WB chimerism |

**Supplemental table 3: Overall and event-free survivals for RAG1/2 and DCLRE1C, stratified by Treosulfan dose**

| **Treosulfan dose** | **RAG1/2 (N=27)** | | | **DCLRE1C (N=10)** | | |
| --- | --- | --- | --- | --- | --- | --- |
|  | **n (%)** | **OS (%)** | **EFS (%)** | **N (%)** | **OS (%)** | **EFS (%)** |
| 30g/m^2^ | 8 (29.6) | 87.5 | 87.5 | 3 (33.3) | 100.0 | 100.0 |
| 36g/m^2^ | 15 (55.6) | 86.7 | 66.7 | 5 (50.0) | 60.0 | 40.0 |
| 42g/m^2^ | 4 (14.8) | 50.0 | 50.0 | 2 (2.0) | 50.0 | 50.0 |
